# Variation of ursolic acid content in flowers of ten Iranian pomegranate (*Punica granatum* L.) cultivars

**DOI:** 10.1186/s13065-019-0598-3

**Published:** 2019-07-03

**Authors:** Fatemeh Sharifiyan, Seyed Abbas Mirjalili, Mohammad Fazilati, Elahe Poorazizi, Saeed Habibollahi

**Affiliations:** 1 Department of Biology, Payame Noor University (PNU), Tehran, Iran; 2 Imam Khomeini Higher Education Center, Agricultural Research, Education and Extension Organization (AREEO), Tehran, Iran; 3 Department of Biochemistry, Najafabad Branch, Islamic Azad University, Najafabad, Iran; 4 Department of Chemistry, Payame Noor University (PNU), Tehran, Iran

**Keywords:** Ursolic acid, Pomegranate flower, HPLC

## Abstract

**Background:**

Ursolic acid (UA) is an important bioactive component in many traditional medicinal plants including pomegranate (*Punica granatum* L.) flower.

**Methods:**

This study presents the HPLC analysis of UA contents of ten cultivars of pomegranate flower grown in Iran. The UA contents of fallen flowers of pomegranate were given in each cultivar.

**Results:**

Remarkable quantities of UA were found in ten cultivars of Iranian pomegranate flower evaluated (21.736 to 15.119 mg/g). Lower quantities of UA were determined in pomegranate fallen flowers (16.763 to 5.754 mg/g).

**Conclusion:**

UA values obtained from Iranian cultivars of pomegranate flowers are very significant when compared with other sources of UA. All of the analyzes suggested that the Iranian pomegranate flowers (including flowers on branches and fallen flowers) might be an excellent UA rich source.

## Introduction

**Fig. 1:**
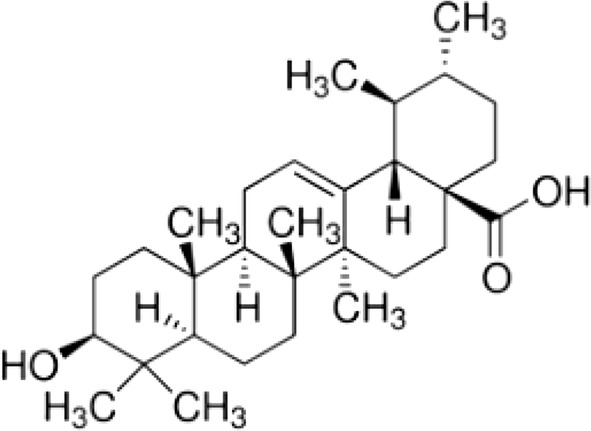
Structure of ursolic acid (UA, 3β-hydroxy-12-urs-12-ene-28-oic acid)

Ursolic acid (UA, 3β-hydroxy-12-urs-12-ene-28-oic acid) (Fig. 1) as an ursane-type pentacyclic triterpene, is a constituent of some medicinal plants [1]. UA possesses considerable pharmacological effects including hepatoprotective [2, 3], immunomodulatory [4], anti-inflammatory [5, 6], antidiabetic [7, 8], antitumor [9, 10], antiulcer [11] and anticancer activities [12, 13]. Recently UA has attracted increasing attention due to its multifunctional anticancer activities [13, 14]. Anti-inflammatory and anti-proliferative, anti-metastatic, proapoptotic, and anti-angiogenic abilities of UA have been reported in both in vivo and in vitro models of cancer [13, 15].

*Punica granatum* Linn. (Punicaceae), commonly known as pomegranate, is a small tree, extensively cultivated in the Middle East, North Africa, the Mediterranean and in parts of Asia [16]. The plant has an immense therapeutic value. Pomegranate, especially its flowers, has been widely used in Ayurvedic, Chinese and Unani medicine systems [17]. Since the flowers are strongly astringent and antidiabetic, a decoction of pomegranate flowers stops bleeding and remedies tympanitis. The flowers are also used in traditional Chinese medicine to cure graying of hair in young men, to treat injuries from falls and to treat chronic diarrhea, especially in children [18, 19]. Pomegranate flowers contain multiple secondary metabolites, the most abundant of which are polyphenols such as gallic acid, ellagic acid and ethyl brevifolin-carboxylate; followed by triterpenes including ursolic, oleanolic, maslinic and asiatic acids. These compounds have shown strong biological activities and medicinal values [20, 21]. As a natural medicine, the type and amount of bioactive ingredients in pomegranate flowers can vary considerably according to the environmental cultivations, soil, water supply, light, geographical origin, the cultivar type used and the time when it is harvested [22]. This study evaluates the variation of UA content in ten cultivars of pomegranate flower grown in Iran, as determined by HPLC analysis. Also in each cultivar, we compared the UA content of flowers picked from branches with fallen flowers.

## Materials and methods

### Chemicals

Ursolic acid standard (European Pharmacopoeia Reference Standard) was purchased from Sigma-Aldrich to control biological and pharmaceutical products. Methanol and water of HPLC grade were obtained from Merck (Germany). Analytical grade Ethanol was purchased from Merck (Germany).

### Plant materials

Flowers of ten Iranian pomegranate cultivars (‘Golnar’, ‘Ghorj Tafti Torsh’, ‘Jangal Sefidrood’, ‘Shirin Bihasteh Mehriz’, ‘Zagh Sefid Yazd’, ‘Alack Zood Ras’, ‘Shirin Siyah Saveh’, ‘Malas Daneh Zard Yazd’, ‘Shirin Shahbar’, ‘Ghojagh Shahpar Varamin’) were collected from Pomegranate trees, which identified originally by a group of botanists, cultivated in the Pomegranate Genotypes Resources Collection (PGRC) subordinate to AREEO (Agricultural Research, Education and Extension Organization, Ministry of Agriculture) in Isfahan Province, Iran in May 2016. In order to compare the UA contents, intact fallen flowers in each cultivar were collected at the same time. The perfect flowers were dried at 105 °C for 15 min, and then at 65 °C for 2 days in a hot-air oven.

### Quantification of UA by HPLC

#### Calibration curve

Stock standard solution of UA was prepared by solving an adequate amount of UA in methanol to obtain an ultimate concentration of 1 mg/mL. A serial dilution was made with methanol to prepare standard solutions at concentrations of 100, 200, 300 and 400 µg/mL, from each of which 20 µL was utilized to plot a standard curve for UA.

#### High-performance liquid chromatography system

Quantification of UA was performed on a SY-8100 system, equipped with SY-8100 HPLC pump, a 7725i manual sample injector, a variable-wavelength UV detector, and SY-8000 HPLC software. The analytical column that was used was Venusil MP C18 (250 mm × 4.6 mm, 5 µm). The isocratic mobile phase was made of methanol and 0.1 M Phosphate buffer (PH = 3, 90:10). The flow rate was 0.9 mL/min and the elute was monitored at 210 nm. The column temperature was kept constant at 21 ± 1 °C. Under these optimized experimental conditions, the HPLC method was applied to analyze the contents of UA in ten cultivars of Iranian pomegranate flower.

#### Preparation of crude extract

In each cultivar, the powder of pomegranate flowers (1 g) was ultrasonically extracted with 20 mL 90% ethanol for 50 min at 40 °C, and then it was filtered. For HPLC analysis, the extracts were passed through a 0.45 µm membrane filter. The peaks related to UA were identified by the retention time and the co-injection test with the standard compound. The UA concentration for all samples was calculated using the peak area based on the standard curve.

#### Precision and recovery studies

To evaluate the intra-day precision of the HPLC method, 200, 300 and 400 µg/mL standard solutions of UA were injected several times (n = 5) over a day. These studies were repeated on different days (n = 5) to evaluate the inter-day precision.

In addition, to test the recovery of the developed method, dried plant powder (1 g) was added with 2 mg UA standard before extraction. Follow-up extraction along with HPLC analysis was performed as described above in detail. The recovery evaluation was as follows:

$$ {\text{Recovery}}\;\left( \% \right) = {{\left( {{\text{A}} - {\text{B}}} \right)} \mathord{\left/ {\vphantom {{\left( {{\text{A}} - {\text{B}}} \right)} {\text{C}}}} \right. \kern-0pt} {\text{C}}} \times 100 $$

In which, A is the result after adding standard, B denotes the amount of sample before adding standard, and C stands for the amount of standard added.

#### Statistical analysis

All data are the mean of three replicates and are presented as mean ± standard deviation (SD). Statistical analysis was performed with the Statistical Analysis System (SAS Institute Inc., Cary, NC) [23].

## Results and discussion

### HPLC separation optimization

Because there are no chromophore moieties in Triterpenoids’ chemical structures, they indicate poor UV absorption, which is the main limitation in analyzing this group of compounds by employing UV detection. In this study, the detection wavelength was chosen at 210 nm for UA because it has better absorption and sensitivity at this wavelength. Based on methanol, acetonitrile, phosphate buffer and phosphoric acid, several mobile phases were tested in order to determine better separation and peak shapes. Finally, the mobile phase consisting of methanol (A) and phosphate buffer (PH = 3) (B) with a ratio of 90:10 (A:B, v/v) was chosen. It was found that simply using methanol separation was unsatisfactory, but as an organic modifier with phosphate buffer solution (pH = 3), methanol performed well. In this regard, adding phosphate buffer improved the separation and peak shapes by controlling pH without ion pairing for acidic compounds. A flow rate of 0.9 mL/min was found appropriate to shorten the run time with no compromise for the peak resolution. The controlled column temperature of 21 ± 1 °C was needed to get reproducible results. The optimized chromatographic conditions were subsequently applied for the analysis of UA content in ten different cultivars of pomegranate flower.

### UA distribution within pomegranate flowers

**Fig. 2:**
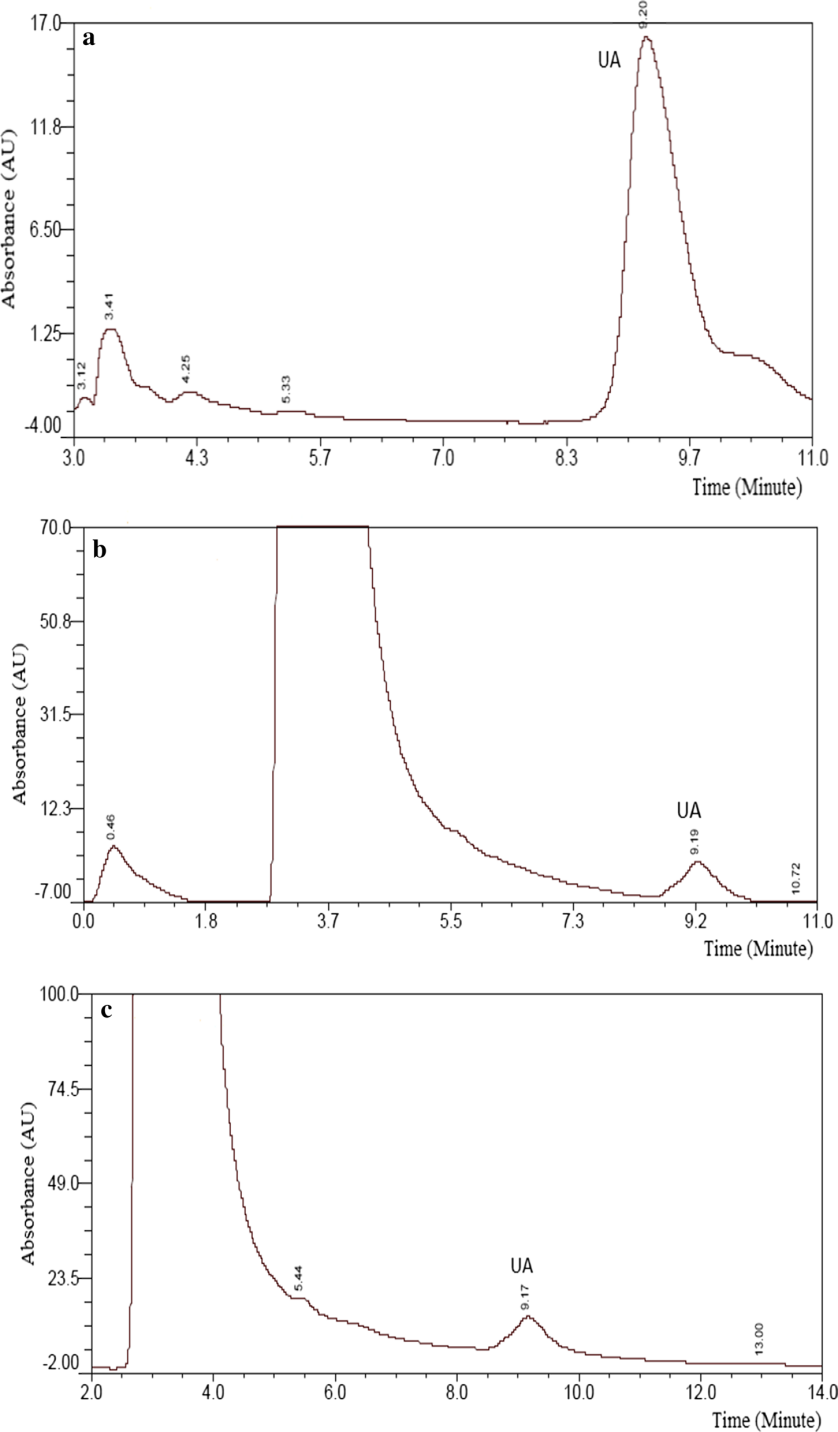
HPLC chromatograms of pomegranate flowers extracts. **a** UA standard; **b** pomegranate flower extract (cv. Shirin Bihasteh Mehriz) (×5); **c** pomegranate fallen flower extract (cv. Shirin Bihasteh Mehriz) (×3). HPLC conditions-column: Venusil MP C18 (250 mm × 4.6 mm, 5 µm); mobile phase: methanol and 0.1 M Phosphate buffer (PH = 3, 90:10); flow rate: 0.9 mL/min; detection wavelength: 210 nm; temperature: 21 ± 1 ^°^C; injection volume: 20 µL

Based on the above detection method, the UA contents in ten cultivars of Iranian pomegranate flowers (including flowers on branches and fallen flowers) were analyzed. The chromatographic retention time for UA was about 9.20 min. The typical chromatograms of the UA standard and extracts of pomegranate flower and pomegranate fallen flower were shown in Fig. 2a–c, which indicate that UA in the pomegranate flower was successfully separated and identified, and that this HPLC system could be used to quantify the content of UA.

The mean values and standard deviations of UA contents in extracts of pomegranate flowers of ten Iranian cultivars are presented in Table 1.

**Table 1:** The UA content in ten cultivars of Iranian pomegranate flower

Cultivar	Crude extract weight (g)^a^	UA contents (mg/g of pomegranate flower dry powder)
Golnar	15.819 ± 0.534	19.514 ± 0.890
Ghorj Tafti Torsh	14.821 ± 0.737	19.044 ± 0.231
Jangal Sefidrood	15.096 ± 0.253	16.268 ± 0.500
Shirin Bihasteh Mehriz	15.769 ± 0.486	21.736 ± 0.564
Zagh Sefid Yazd	15.120 ± 0.698	19.089 ± 0.962
Alack Zood Ras	15.237 ± 0.298	20.294 ± 0.306
Shirin Siyah Saveh	14.968 ± 0.894	20.517 ± 0.855
Malas Daneh Zard Yazd	15.538 ± 0.773	21.622 ± 1.00
Shirin Shahbar	15.458 ± 0.163	19.063 ± 0.486
Ghojagh Shahpar Varamin	15.775 ± 0.463	15.119 ± 0.663

Data are means (n = 3) ± standard deviation

^a^The weight of the extract is related to the liquid extract

The content of UA varied significantly (P < 0.01) in the cultivars of Iranian pomegranate flowers analyzed in this study. UA contents ranged from 21.736 to 15.119 mg of ursolic acid/g pomegranate flower dry powder, being highest in ‘Shirin Bihasteh Mehriz’ and lowest in ‘Ghojagh Shahpar Varamin’ cultivar. The average UA content of ten pomegranate flower cultivars was 19.226 (mg/g) significantly depending on the cultivar. High UA levels were found in ‘Shirin Bihasteh Mehriz’ (21.736 mg/g), ‘Malas Daneh Zard Yazd’ (21.622 mg/g), ‘Shirin Siyah Saveh’ (20.517 mg/g), ‘Alack Zood Ras’ (20.294 mg/g), ‘Golnar’ (19.514 mg/g), ‘Zagh Sefid Yazd’ (19.089 mg/g), ‘Shirin Shahbar’ (19.06 mg/g) and ‘Ghorj Tafti Torsh’ (19.044 mg/g), but in ‘Jangal Sefidrood’ (16.268 mg/g) and ‘Ghojagh Shahpar Varamin’ (15.119 mg/g), lower levels of UA were observed. Variations in the range of UA values observed in this study could be due to differences in cultivars used. Such variations can also suggest a variety of ecological functions throughout evolution. These possible functions include interactions between plants and herbivorous insects. UA is variously reported to possess anti-insect activity [24–26]. González-Coloma et al. [27] evaluated the general activity of pentacyclic triterpenes such as ursane and lupane, and explained their antifeedant effects on several pest insects (*Leptinotarsa decemlineata, Myzus persicae* and *Spodoptera littoralis*). In another study, of the five compounds isolated from the leaf extracts of *Vitex negundo* L., ursolic acid showed more effective antifeedant activity against the third instar larvae of castor semilooper [24]. Therefore, along with other biological activities of pentacyclic triterpenes, UA is probably related to its role in plant defenses against insects; hence, probably, cultivars of pomegranate flower with a higher UA content have been more exposed to herbivorous insects than those with less UA.

In order to compare the collected results with the UA values in pomegranate fallen flowers, the UA contents of fallen flowers in each cultivar are presented in Table 2.

**Table 2:** The UA content in ten cultivars of Iranian pomegranate fallen flowers

Cultivar	Crude extract weight (g)^a^	UA contents (mg/g of pomegranate flower dry powder)
Golnar	14.895 ± 0.436	9.269 ± 0.378
Ghorj Tafti Torsh	14.655 ± 0.837	12.153 ± 0.399
Jangal Sefidrood	15.104 ± 0.859	12.755 ± 0.009
Shirin Bihasteh Mehriz	15.669 ± 0.383	15.332 ± 0.548
Zagh Sefid Yazd	14.242 ± 0.533	5.754 ± 0.435
Alack Zood Ras	15.311 ± 0.728	11.755 ± 0.826
Shirin Siyah Saveh	14.983 ± 0.786	16.763 ± 0.865
Malas Daneh Zard Yazd	15.348 ± 0.637	10.042 ± 0.332
Shirin Shahbar	15.934 ± 0.845	15.763 ± 0.304
Ghojagh Shahpar Varamin	15.665 ± 0.355	6.658 ± 0.586

Data are means (n = 3) ± standard deviation

^a^The weight of the extract is related to the liquid extract

UA was detected in the fallen flowers of all pomegranate cultivars analyzed in this study. Statistically, there were significant differences (P < 0.01) between the UA contents of fallen flowers in different cultivars. For the fallen flowers, UA contents ranged from 16.763 to 5.754 mg of Ursolic acid/g pomegranate flower dry powder, for cultivars of Shirin Siyah Saveh and Zagh Sefid Yazd, respectively. The average UA content of ten cultivars of pomegranate fallen flowers was 11.624 (mg/g). In each cultivar, the determined values of UA in pomegranate fallen flowers were remarkably lower than those in flowers picked from branches, on the average 1.65 times. These decreased levels of UA observed in fallen flowers probably happened after flowers fell from the trees and were related to environmental effects.

Researchers have determined UA content in several different plants, including *Ligustrum lucidum* Ait. (9.8 mg/g) [28], *Eriobotrya japonica* Lindl. (5.6 mg/g) [29], *Rosmarinus officinalis* leaves (15.8 mg/g) [30] and *Ziziphora clinopodioides* Lam. (1.176 mg/g) [31]. Jager et al. [32] quantified the triterpene content of 39 plant material. They determined maximum concentration of UA in *Rosmarinus officinalis* leaves (29.5 mg/g), *Salvia officinalis* leaves (18 mg/g), *Coffea arabica* leaves (18 mg/g), *Lavandula angustifolia* leaves (15.9 mg/g) and *Malus domestica* peels (14.3 mg/g). Although UA is present in a wide variety of plants, our results showed that Iranian pomegranate flowers (including flowers on branches and fallen flowers) are good sources of UA.

### Linearity, precision and recovery of the HPLC method

The linearity of the responses from the detector was studied for standard substance by plotting peak areas against the injected values. There was good agreement between the peak area and the standard values in the range of 100–400 µg/mL for UA. The regression equation and coefficient of determination were [y = 0.0006x + 18.142] (R^2^ = 0.9955) for UA.

The inter-day and intra-day variations for the determination of UA were less than 3% at concentrations of 200, 300 and 400 µg/mL (Table 3). The low values of %RSD show the high accuracy of the method.

**Table 3:** Inter-day and intra-day precision for UA

Concentration (µg/mL)	RSD%	
	Inter-day (n = 5)	Intra-day (n = 5)
200	2.21	1.56
300	1.99	1.63
400	1.51	2.13

By mixing a suitable amount of quantified samples with the standard compound, recovery experiment was conducted in order to confirm that the method was accurate, and the average recovery of UA was 100%.

## Conclusion

In this study, the investigation of UA contents of pomegranate flowers from ten cultivars grown in Iran was performed. Among the ten pomegranate flower cultivars researched, eight cultivars including ‘Shirin Bihasteh Mehriz’, ‘Malas Daneh Zard Yazd’, ‘Shirin Siyah Saveh’, ‘Alack Zood Ras’, ‘Golnar’, ‘Zagh Sefid Yazd’, ‘Shirin Shahbar’ and ‘Ghorj Tafti Torsh are characterized by the highest content of UA and can be used as a rich source of UA for the synthesis of a wide variety of bioactive compounds such as food supplements and health products. However the results of this study showed that the UA content of pomegranate flowers is significantly reduced after falling from trees, but due to the high content of UA in pomegranate flowers and considering the fact that a large number of flowers of pomegranate trees fall and are discarded by farmers, the results suggested that the pomegranate fallen flowers are valued as a source of UA.

## Data Availability

The datasets used and analysed during the current study are available from the corresponding author on reasonable request.

## Abbreviations

cv.: cultivar
HPLC: high performance liquid chromatography
UA: ursolic acid
